# Maize lethal necrosis (MLN): Efforts toward containing the spread and impact of a devastating transboundary disease in sub-Saharan Africa

**DOI:** 10.1016/j.virusres.2020.197943

**Published:** 2020-06

**Authors:** Prasanna Boddupalli, L.M. Suresh, Francis Mwatuni, Yoseph Beyene, Dan Makumbi, Manje Gowda, Mike Olsen, David Hodson, Mosisa Worku, Monica Mezzalama, Terence Molnar, Kanwarpal S. Dhugga, Anne Wangai, Lilian Gichuru, Samuel Angwenyi, Yoseph Alemayehu, Jens Grønbech Hansen, Poul Lassen

**Affiliations:** a International Maize and Wheat Improvement Center (CIMMYT), ICRAF Campus, UN Avenue, Gigiri, Nairobi, Kenya; b CIMMYT, Km 45 México-Veracruz, El Batán, 56237, Texcoco, Mexico CDMX, Mexico; c Kenya Agricultural and Livestock Research Organization (KALRO), NARL, Waiyaki Way, Nairobi, Kenya; d Alliance for Green Revolution in Africa (AGRA), West End Towers, 4th Floor Kanjata Road, off Muthangari Drive, Off Waiyaki Way, P.O. Box 66773, Westlands, 00800, Nairobi, Kenya; e African Agricultural Technology Foundation (AATF), ILRI Campus, Naivasha Road, Nairobi, Kenya; f CIMMYT, ILRI campus, P.O. Box 5689, Addis Ababa, Ethiopia; g Dept. of Agroecology, Aarhus University, Blichers Allé 20, Postboks 50, DK-8830, Tjele, Denmark

**Keywords:** CIMMYT, International Maize and Wheat Improvement Center, KALRO, Kenya Agriculture and Livestock Research Organization, MABC, Marker-assisted backcrossing, MCMV, maize chlorotic mottle virus, MLN, Maize lethal necrosis, NARS, National Agricultural Research System, NPPO, National Plant Protection Organization, QTL, Quantitative Trait Locus, SCMV, sugarcane mosaic virus, SNP, Single nucleotide polymorphism, Maize lethal necrosis, Maize chlorotic mottle virus, Africa, Breeding, Diagnostics, Surveillance

## Abstract

Maize lethal necrosis (MLN), a complex viral disease, emerged as a serious threat to maize production and the livelihoods of smallholders in eastern Africa since 2011, primarily due to the introduction of maize chlorotic mottle virus (MCMV). The International Maize and Wheat Improvement Center (CIMMYT), in close partnership with national and international partners, implemented a multi-disciplinary and multi-institutional strategy to curb the spread of MLN in sub-Saharan Africa, and mitigate the impact of the disease. The strategy revolved around a) intensive germplasm screening and fast-tracked development and deployment of MLN-tolerant/resistant maize hybrids in Africa-adapted genetic backgrounds; b) optimizing the diagnostic protocols for MLN-causing viruses, especially MCMV, and capacity building of relevant public and private sector institutions on MLN diagnostics and management; c) MLN monitoring and surveillance across sub-Saharan Africa in collaboration with national plant protection organizations (NPPOs); d) partnership with the private seed sector for production and exchange of MLN pathogen-free commercial maize seed; and e) awareness creation among relevant stakeholders about MLN management, including engagement with policy makers. The review concludes by highlighting the need to keep continuous vigil against MLN-causing viruses, and preventing any further spread of the disease to the major maize-growing countries that have not yet reported MLN in sub-Saharan Africa.

## Highlights

• Maize lethal necrosis (MLN) emerged as a serious threat to maize production and livelihoods of smallholders in eastern Africa since 2011.
• An intensive multi-disciplinary and multi-institutional strategy is being implemented to curb the spread of MLN in sub-Saharan Africa, and mitigate the impact of the disease.
• Intensive germplasm screening led to identification of MLN-resistant sources, and fast-tracked development and commercial release of 19 MLN-tolerant/resistant hybrids in eastern Africa.
• Marker-assisted breeding led to successful conversion of 52 elite but MLN-susceptible inbred lines into MLN-resistant versions.
• MLN/MCMV diagnostic protocols have been optimized, and personnel from relevant public and private sector institutions trained on MLN diagnostics, monitoring and surveillance.

## 1 Introduction

Maize (*Zea mays* L.) is the most important cereal crop in sub-Saharan Africa (SSA), covering over 35 million hectares, largely in smallholder farming systems that produce over 70 million metric tons of grain (FAO, 2018). The crop is critical for food security, income and livelihoods of several million smallholders across SSA, and especially in eastern and southern Africa, where nearly 85% of the maize produced is used as food (Shiferaw et al., 2011). However, average maize yield in SSA (∼1.7 tons/hectare) is far below the global average (∼5 tons/hectare), for various reasons, including frequent occurrence of drought, poor soil fertility, inadequate availability and use of inputs (improved seed and fertilizer), and pest and disease challenges.

The spread of transboundary pests and diseases has increased significantly in recent years, affecting food and income security of several million resource-poor farmers, especially in SSA, Asia and Latin America. Globalization, trade and climate change, as well as reduced resilience in production systems due to decades of agricultural intensification, are the contributing factors. A major example is the emergence of maize lethal necrosis (MLN) in East Africa, which was first reported in the southern Rift Valley of Kenya in 2011, and then soon after in other eastern African countries during 2012–2014 (Wangai et al., 2012; Adams et al., 2014; Lukanda et al., 2014; Mahuku et al., 2015a, b; Redinbaugh and Stewart, 2018). MLN is a disease caused by combined infection of maize plants with maize chlorotic mottle virus (MCMV; genus *Machlomovirus*, family *Tombusviridae*) (Niblett and Claflin, 1978), with any one of several viruses from the family *Potyviridae*, such as sugarcane mosaic virus (SCMV), maize dwarf mosaic virus (MDMV) or wheat streak mosaic virus (WSMV) (Redinbaugh and Stewart, 2018). While SCMV has worldwide distribution that includes SSA, the outbreak of MLN in Africa was driven by the emergence and spread of MCMV.

The dynamics of the MLN epidemic in eastern Africa is complex. In addition to the newly introduced MCMV and endemic potyviruses such as SCMV, other members of the family *Potyviridae* have been reported to cause MLN in the region. Recently, a distinct isolate of Johnsongrass mosaic virus (JGMV) was found to be widespread in the region and could be associated with MLN (Stewart et al., 2017). The epidemiology of MLN, including insect vectors of MCMV and potyviruses, seed as a source of infection, and virus transmission through soil, was discussed in detail by Redinbaugh and Stewart (2018).

## 2 Global occurrence and impact of MLN

MCMV was first reported in Peru in 1974 (Castillo and Hebertt, 1974). MLN was subsequently found in the USA (Niblett and Claflin, 1978). The disease was later reported from several countries across the Americas, Asia, and Africa, including Argentina (Teyssandier et al., 1982), Thailand (Klinkong and Sutabutra, 1982; Uyemoto, 1983), Mexico (Delgadillo and Gaytán, 1987), China (Xie et al., 2011), Kenya (Wangai et al., 2012), Uganda (Mahuku et al., 2015a,b), Rwanda (Adams et al., 2014), D.R. Congo (Lukanda et al., 2014), Ethiopia (Mahuku et al., 2015b), Taiwan (Deng et al., 2014), Ecuador (Quito-Avila et al., 2016), and Spain (Achon et al., 2017). After the first detection of MLN in Kenya in September 2011, followed by confirmation of the pathogens involved in the disease in Africa (Wangai et al., 2012), MCMV was recorded across the region over the next 3–4 years.

MLN had a serious impact on maize production and grain yields in eastern Africa (De Groote et al., 2016; Marenya et al., 2018). During 2012–2013, the estimated maize yield losses in Kenya due to MLN were reported as 23–100% in the affected counties in the country. In 2012, the estimated losses due to MLN were up to US$ 52 million, and by 2013 (De Groote et al., 2016) it was estimated that the aggregate national loss of maize production due to MLN was about 0.5 million tons with a value of US$ 180 million. The disease is still a major threat to the maize crops in eastern Africa (Isabirye and Rwomushana, 2016), and the threat of its emergence in other regions in SSA still looms.

MLN had a devastating effect not only on the maize crop and the livelihoods of the resource-poor farmers in the affected countries, but also on other key actors in the maize seed/grain value chain, especially small- and medium-enterprise (SME) seed companies and processors. Demand for seed of commercial maize varieties decreased when MLN was a major epidemic in the affected countries, with consequent losses of sales for maize-based seed companies, and carry-over of significant quantities of seed. Thus, in addition to resource-poor farmers, small- and medium-enterprise seed companies were highly affected by the intensity and spread of MLN in eastern Africa.

Effectively countering the incidence, spread and adverse impacts of MLN in Africa requires strong, coordinated and synergistic efforts from multiple institutions as the challenge is complex and multi-faceted. Since 2012, the International Maize and Wheat Improvement Center (CIMMYT) team in Africa, in partnership with Kenya Agriculture and Livestock Research Organization (KALRO), National Plant Protection Organizations (NPPOs), commercial seed companies, several advanced research institutions in the USA and Europe, International Institute of Tropical Agriculture (IITA), non-government organizations, such as Alliance for Green Revolution in Africa (AGRA) and African Agricultural Technology Foundation (AATF), have been intensively implementing a multi-disciplinary and multi-institutional strategy for curbing the spread and limiting the impact of MLN in Africa.

The strategy revolved around a) fast-tracked development and deployment of MLN-tolerant/resistant maize hybrids in Africa-adapted genetic backgrounds; b) diagnostics for MLN-causing viruses; c) MLN monitoring and surveillance across Africa; d) production and exchange of MLN pathogen-free commercial maize seed; d) engagement with policy makers; and e) awareness creation among relevant stakeholders about MLN management, including establishment of an MLN phytosanitary community of practice.

## 3 Breeding for MLN resistance in Africa-adapted maize germplasm

Studies during 2012-13 confirmed that nearly all commercial maize varieties in Kenya were susceptible to MLN, both under natural and artificial infection conditions (Prasanna, 2015; Marenya et al., 2018). Severe MLN infection in farmers’ fields was found to cause up to 100% yield loss in susceptible varieties (Mahuku et al., 2015a). Development of MLN-tolerant/resistant maize varieties is, therefore, the most economically viable and environmentally sustainable approach, which requires intensive screening of germplasm, identification of resistant genotypes, and incorporation of MLN resistance in combination with other relevant traits into suitable genetic backgrounds. All this had to be done in an accelerated manner so that improved varieties with MLN tolerance/resistance were released in affected countries, and farmers were able to access the seed of such varieties.

In partnership with KALRO, CIMMYT established a centralized MLN Quarantine and Screening Facility at KALRO Research Center at Naivasha (Kenya) in September 2013. The 20-hectare facility, established with financial support from the Bill & Melinda Gates Foundation (BMGF) and Syngenta Foundation for Sustainable Agriculture (SFSA), includes 17 ha for field screening under MLN artificial inoculation, an MLN diagnostics laboratory, nearly 2000 m^2^ of greenhouses, 3500 m^2^ of net houses (for separate screening for responses to MCMV or SCMV under artificial inoculation), etc. MLN phenotyping is carried out throughout the year (two times a year for field-based MLN screening, and thrice per year for MLN indexing). The facility presently has the capacity to screen at least 82,000 germplasm entries or genotypes per year under artificial inoculation, with uniform disease pressure across field trials, producing high-quality data on the response of maize germplasm against MLN.

The MCMV and SCMV isolates used at the MLN Screening Facility were initially collected from maize fields in MLN hot spot areas in Kenya. The mother cultures of SCMV and MCMV are maintained on susceptible maize hybrids (H614 and PBG30G19) under isolated greenhouse conditions, and regularly verified using enzyme-linked immune sorbent assay (ELISA). Plants for inoculum increase are inoculated at 4-5-leaf stage and leaves from inoculated plants are used as inoculum sources. MCMV/SCMV inoculum production for screenhouse trials as well as MLN inoculum (with both MCMV and SCMV) production for field trials were optimized (Mahuku et al., 2015a; Gowda et al., 2018; https://mln.cimmyt.org/). Inoculum for the MLN field trials is prepared by preparing an optimized combination of SCMV and MCMV viruses (ratio of 4:1). The protocols for MCMV and SCMV culture maintenance, MLN inoculum preparation, and artificial inoculation were described earlier (Gowda et al., 2015; Sitonik et al., 2019).

After several experiments, an efficient and cost-effective phenotyping system, including MLN virus inoculation and disease scoring, was developed (Semagn et al., 2015; Mahuku et al., 2015a; Beyene et al., 2017; https://mln.cimmyt.org/), leading to identification of genotypes with resistance to MLN/MCMV/SCMV, and incorporation of these genotypes into breeding pipelines. Inoculation is carried out twice at one-week intervals to reduce any possibility of escapes. MLN disease severity is visually scored on each plot in an ordinal scale of 1 (highly resistant, with no disease symptoms) to 9 (highly susceptible, with severe necrosis and death). Data were recorded at 10 day intervals, beginning from 10 days after second inoculation, up to 3–4 times. The final disease severity score is recorded 40 days after first inoculation. For germplasm classification, mean MLN severity scores between 7 and 9 are considered “susceptible”, scores of 5 or 6 as “tolerant”, and scores between 1 and 4 as “resistant”. In addition to MLN severity score (based on disease symptoms), yield penalty (i.e., grain yield under no MLN infection versus yield under artificial inoculation) is also considered for rating a hybrid as MLN-tolerant or MLN-resistant.

Native genetic “resistance” to plant viruses, including MLN-causing viruses, cannot be “complete” i.e., the plants are neither immune nor do they completely inhibit the replication of the disease-causing viruses. Nevertheless, it must be recognized that developing and deploying elite maize varieties with tolerance/resistance to MLN-causing viruses (including MCMV) offers an effective and important practical solution against MLN, as compared to commercializing MLN-susceptible varieties extensively in the MLN-prevalent countries. Studies done by the CIMMYT team on MCMV titers in MLN-resistant vs MLN-tolerant vs MLN-susceptible genotypes revealed statistically significant differences, with MLN-resistant and MLN-tolerant genotypes showing on average ∼68% and ∼47% lower MCMV titers compared to the MLN-susceptible (L.M. Suresh, unpublished data).

**Fig. 1:**
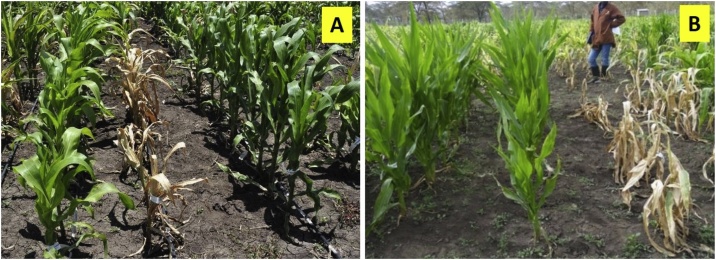
**(A)** MLN-resistant inbred lines, CKDHL1705340 (left) and CKDHL1705309 (right), flanking an MLN-susceptible line, CML442; **(B)** MLN-resistant line, KS23-6 (left) versus an MLN-susceptible line, CML547 (right), 30 days after first artificial inoculation at the MLN Screening Facility, Naivasha (Kenya).

Breeding for MLN resistance is now an integral component of product-profile based maize breeding, especially at CIMMYT’s eastern Africa hub at Kenya since 2012. This includes routine screening of breeding materials in various stages under MLN artificial inoculation at the Naivasha facility; identification of resistance sources from diverse germplasm (Fig. 1); accelerated breeding using doubled haploids (DH) technology and molecular markers; stage-gate product advancement, and varietal release and deployment of elite MLN tolerant/resistant hybrids through public and private sector partners.

### 3.1 Extensive germplasm screening and development of MLN-tolerant/resistant hybrids in Africa

The first screening of commercial maize varieties, including hybrids and open-pollinated varieties (OPVs), marketed in Kenya showed high levels of susceptibility to MLN under artificial inoculation (Prasanna, 2015; Semagn et al., 2015). Since 2013, CIMMYT and partners screened nearly 200,000 genotypes (∼300,000 rows of 3 m length each) at the MLN screening facility in Naivasha under artificial inoculation. Of these, 63% were from CIMMYT, 16% were from NARS institutions, and 21% from private sector (https://mln.cimmyt.org/). The efficiency of MLN phenotyping under artificial inoculation at Naivasha could be gauged from the “heritability” values of MLN trials conducted since 2014. “Heritability” is a statistic that estimates the degree of variation in a phenotypic trait in a population that is due to genetic variation among individuals in that population. For MLN phenotyping at Naivasha, the heritability values across various trials ranged from 0.71 to 0.95, with a mean heritability of 0.83.

More than 3000 lines derived from both pedigree and doubled haploid (DH) technology by CIMMYT’s tropical breeding programs and adapted from the lowlands to highlands were genotyped using genotyping-by-sequencing (GBS) SNPs (Gowda et al., 2015, 2018; Sitonik et al., 2019; Nyaga et al., 2020). Diversity analyses based on >300,000 high quality GBS SNPs revealed a high variation in relatedness among these lines. The genetic distances among these 3000 inbred lines ranged from 0 to 0.65, with a mean of 0.39 (Manje Gowda, unpublished data). The same marker data is used in the CIMMYT studies on discovery and validation of genomic regions influencing MLN/MCMV resistance in maize.

**Fig. 2:**
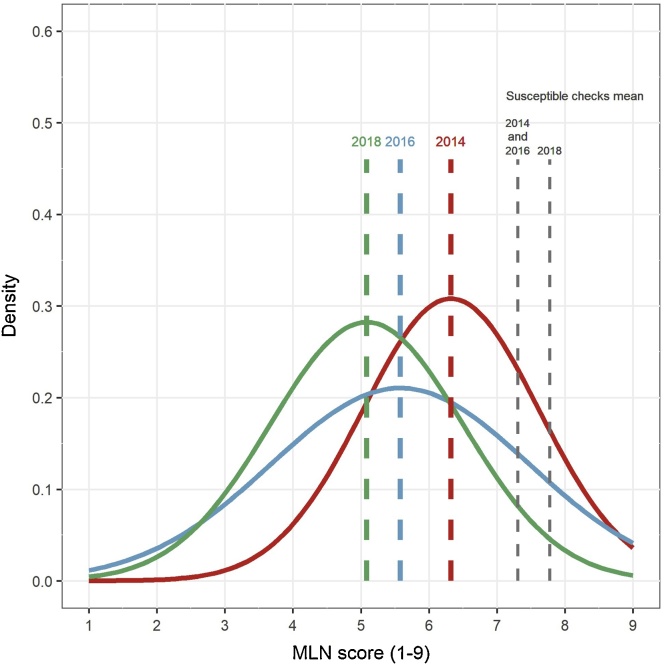
Frequency distribution of maize lines (n = 5307) derived during 2014–2018 for MLN severity scores, based on evaluation under MLN artificial inoculation at Naivasha in Kenya.

CIMMYT followed conventional pedigree breeding and molecular marker-assisted breeding methods to develop maize germplasm with resistance to MLN. To analyze the progress made in breeding for resistance to MLN, in 2019, a total of 5307 inbred lines (2876 lines developed in 2014; 1522 lines developed in 2016; 909 lines developed in 2018) were evaluated in an experiment at the MLN screening facility at Naivasha under artificial inoculation in replicated trials. The differential response of the lines was striking among the MLN-resistant and susceptible lines at Naivasha. We observed a considerable number of lines susceptible to MLN among the 2014 lines (with a mean score of 6.4), followed by lines from 2016 (with a mean score of 5.7), while in the lines from 2018, the mean MLN score was 5.1; the susceptible inbred checks had a mean MLN severity score of 7.3 in the year 2014 and 2016, and 7.8 in the year 2018 (Fig. 2). This demonstrated significant breeding progress made within a short period of time. This breeding achievement could be attributed to: 1) crossing among MLN-tolerant lines identified from the previous year screening; 2) creation of a large number of DH lines per population to ensure the likely occurrence of rare genetic combinations; and 3) optimized artificial screening protocols that allowed effective identification of MLN-resistant lines.

CIMMYT, in collaboration with NARS partners, also created a network of MLN screening sites (at MLN hot spots with high natural disease pressure) in Kenya, Tanzania, Uganda and Ethiopia, for evaluating promising pre-commercial MLN-tolerant/resistant hybrids identified at the MLN screening facility at Naivasha. Through this network, promising pre-commercial hybrids with MLN tolerance/resistance, coupled with desirable agronomic traits, including good husk cover, reduced ear rots, and tolerance to other stresses were identified.

Through intensive efforts, four first-generation CIMMYT-derived MLN-tolerant hybrids (with a mean MLN severity score of 5.5 on a 1–9 scale) were initially released in MLN-prevalent East Africa (1 hybrid in Uganda; 2 in Kenya; 1 in Tanzania) through partners during 2013–2014. Subsequently, several second-generation MLN-resistant hybrids (with MLN severity scores of 4.0 or less than 4.0 on a 1–9 scale) have been developed by CIMMYT and released in East Africa in collaboration with public and private sector partners. By 2019, a total of 18 MLN-tolerant/resistant hybrids had been released in East Africa (Table 1). Combined analysis of data generated over 18 first-generation and 19 second-generationn MLN-tolerant/resistant hybrids (including precommercial and commercial) along with 11 commercial MLN-susceptible hybrids (non-CIMMYT) across two years (2016 and 2017) and with two simultaneous trials each year – a) under MLN artificial inoculation at the MLN screening facility in Naivasha; and b) under no MLN infection at Naivasha (outside the screening facility) showed significant differences in MLN severity scores, grain yield, and yield penalty (yield under MLN artificial inoculation versus no infection) among the three groups of hybrids (Table 2). While the mean grain yield of the commercial MLN-susceptible hybrids was 0.7 t/ha with an yield penalty of 77.7% under artificial inoculation, the first-generation and second-generation hybrids recorded 3.4 t/ha and 4.6 t/ha, with yield penalty of 22.9% and 3.1%, respectively.

**Table 1:** List of CIMMYT-derived MLN-tolerant hybrids released till 2019 in East Africa.

S.No.	Hybrids	Year of Release	Country	Partner organization that released the hybrid*	MLN severity score (on 1-9 scale)**	First- or second-generation ML**N-**tolerant hybrid?
1	H6505	2013	Kenya	KSC	6	First-generation
2	Bazooka	2014	Uganda	NASECO	5	First-generation
3	H6506	2014	Kenya	KSC	6	First-generation
4	Meru HB607	2014	Tanzania	Meru Agro Seed Company	6	First-generation
5	WE5135	2016	Kenya; Tanzania	KALRO (Kenya); TOSCI (Tanzania)	4	Second-generation
6	WE5140	2016	Kenya	KALRO	4	Second-generation
7	WE6109	2016	Kenya	KALRO	4	Second-generation
8	WE6110	2016	Kenya	KALRO	4	Second-generation
9	KATEH16-01	2017	Kenya	KALRO	4	Second-generation
10	KATEH16-02	2017	Kenya	KALRO	3	Second-generation
11	KATEH16-03	2017	Kenya	KALRO	4	Second-generation
12	WHMLN	2017	Kenya	Western Seed Company	4	Second-generation
13	WE7117	2018	Kenya	KALRO	4	Second-generation
14	WE7118	2018	Kenya	KALRO	3	Second-generation
15	WE7119	2018	Kenya; Tanzania	KALRO (Kenya); TOSCI (Tanzania)	4	Second-generation
16	WE5141	2019	Tanzania	COSTEC	4	Second-generation
17	WE7133	2019	Tanzania	COSTEC	4	Second-generation
18	CKMLN150074	2019	Kenya	Seed Co. Ltd	4	Second-generation

* KSC: Kenya Seed Company; KALRO: Kenya Agriculture and Livestock Research Organization; TOSCI: Tanzania Official Seed Certification Institute.

** Average score based on several trials undertaken at the MLN Screening Facility, Naivasha, during 2013-2019. Note that mean severity score of the MLN-susceptible commercial checks in eastern Africa is 7.5, as recorded in the trials at Naivasha during 2013-2019.

**Table 2:** Performance of the first-generation and second-generation MLN-tolerant/resistant hybrids vis-à-vis commercial MLN-susceptible hybrids under MLN artificial inoculation and without MLN inoculation (control) at Naivasha, Kenya during 2016 and 2017.

Category	No. of hybrids evaluated	Mean grain yield (t/ha) without MLN infection	Mean grain yield (t/ha) und MLN artificial inoculation	Mean MLN severity score (on a 1-9 scale)	Mean grain yield loss due to MLN (%)
Commercial MLN-susceptible hybrids	11	3.0	0.7	6.8	77.7
First-generation MLN tolerant precommercial and commercial hybrids	18	4.4	3.4	5.0	22.9
Second-generation MLN-resistant precommercial and commercial hybrids	19	4.7	4.6	3.9	3.1

To analyze the genetics of MLN resistance in CIMMYT’s maize germplasm in Africa, Beyene et al. (2017) conducted a diallel experiment of 340 single-cross hybrids and four commercial checks for two years under artificial MLN inoculation, and reported that additive gene action is more important than non-additive gene action for MLN resistance; thus, rapid progress could be expected from recurrent selection. The study also identified MLN-tolerant inbred lines and single-cross hybrids that could be used in breeding programs to develop elite, MLN-tolerant/resistant hybrids in SSA. Further prediction of hybrid performance for MLN resistance with mid-parent value was moderate to low but improved significantly with general combining ability (GCA) and/or molecular markers (unpublished results). Prediction of hybrid performance for MLN resistance based on mid-parent value was low with R^2^ = 0.27 whereas the prediction accuracy was 0.80 based on GCA. This clearly indicates that MLN resistance is mainly controlled by genetic effects, and therefore, it is possible to select the best hybrid combination with MLN resistance solely based on prior information of the GCA effects of the lines (Beyene et al., 2017).

### 3.2 Pre-breeding for MCMV resistance

In evaluating a large collection of maize germplasm materials for MLN resistance at the MLN screening facility (see above section), it became apparent that very few of the elite lines used in public and private sector breeding programs have resistance to MLN, and more specifically, MCMV (Beyene et al., 2017; Gowda et al., 2015). To address this lack of diversity for MCMV resistance, the CIMMYT Genetic Resources Program in Mexico launched an evaluation of maize landraces from the CIMMYT Germplasm Bank (CGB) collection, with the goal of discovering germplasm with potentially novel haplotypes for MLCMV resistance. The CGB has over 27,000 unique maize germplasm accessions, the majority of which are landraces; it would be an overwhelming challenge financially and logistically to screen all of them for their responses to MCMV. Therefore, the collection was stratified to identify those accessions with a higher likelihood of having MCMV resistance. The first stratification made was geographic, focusing on Latin America, an area that contains a vast amount of maize genetic variation, with Mexico being the center of diversity for over 300 described races (Goodman and Brown, 1988; Vigouroux et al., 2008; Wellhausen et al., 1952). Additionally, Latin America is endemic for many maize viral diseases, including MCMV, that have coevolved with maize for at least 2000 years (Brewbaker, 1979; Nault, 1979; Redinbaugh et al., 2004). For the second stratification, we used a combination of landrace passport data and genetic data to make a final selection of 1000 landraces for the study. Passport data includes racial classification for each of the landrace collections, including the longitude, latitude, altitude and the village at which the collection was made. The passport data allowed us to select landraces from a wide variety of races, geographically dispersed locations and a range of altitudes from within Latin America. The genetic data comes from high density SNP genotypic data generated across the CGB landrace accessions by the Seeds of Discovery Project (http://hdl.handle.net/11529/10548358; https://seedsofdiscovery.org/). The genetic data allowed us to select accessions based on an optimized sampling of genetic space using genomic distance, as described in Franco et al. (2005) and Franco-Duran et al. (2019). The final 1000 selected landraces were from 25 countries and represented 92 different maize races (Table 3).

**Table 3:** Number of landraces selected from different Latin American countries for their responses to MCMV infection.

Country/Region	Selected	Races*
Mexico	435	36
Guatemala	150	21
Venezuela	117	13
Peru	108	20
Caribbean Islands	115	11
Ecuador	36	6
Colombia	29	8
Guyanas	6	3
Argentina	4	1
**Total**	**1000**	**119**

* Because some races are regional (e.g., Tuxpeño) the total number of unique races was 92 (not 119).

Beginning in 2015, the 1000 selected landraces were evaluated for their responses to MCMV under artificial inoculation in replicated trials in the Biosafety Greenhouses at CIMMYT, Mexico. A Kenyan isolate of MCMV was used to carry out the mechanical inoculation of maize seedlings following the afore mentioned protocols. Every plant of each replication was scored individually because maize landraces are genetically heterogenous and thus each plant is a unique genotype. In these initial evaluations the goal was to identify, out of the 1000 landraces, a subset of landraces with MCMV resistance. Simultaneous with the MCMV evaluations, each of the landrace accessions were crossed in a nursery to one of four selected elite CIMMYT maize lines or CMLs, and subsequently self-pollinated twice to the F_3_ generation, with a goal of producing at least 50 F_3_ lines per population. While 11 landraces exhibited putative resistance to MCMV, relative to CML494 and CML550, only nine produced a sufficient number of F_3_ lines for greenhouse evaluation (Table 4). A total of 738 F_3_ lines were evaluated in the greenhouse for MCMV resistance, and based on the F_3_ line evaluations, 255 F_4_ lines derived from the best F_3_ lines were shipped in late 2017 to the MLN screening facility in Naivasha, Kenya, for advanced MCMV evaluation in the screenhouses. Additionally, the F_3_ lines were genotyped. The phenotypic and genotypic data are being analyzed to identify novel haplotypes for MCMV resistance. Currently, 118 F_5_ lines derived from the best F_4_ lines are being evaluated for MLN in the field in Naivasha and will be further evaluated for their yield and other relevant agronomic traits. Although incorporation of chromosome segments from useful landrace germplasm into elite lines and hybrids is a medium- to long-term endeavor, appropriate funding and strong planning has allowed for relatively rapid identification of MCMV-tolerant germplasm and development of bridging germplasm lines for potential use in elite CIMMYT breeding programs in Kenya. One clear conclusion from the data generated in the maize landrace evaluation work is that germplasm from the Caribbean region is rich in alleles for MCMV resistance. Of the 11 landrace accessions identified with putative MCMV resistance, 6 (55%) were from the Caribbean region, while the Caribbean landraces comprised only 12% of the 1000 landraces that were initially evaluated (Table 3).

**Table 4:** Eleven maize landrace accessions selected for continued breeding based on greenhouse evaluations at CIMMYT-Mexico for MCMV resistance.

Entry	Country	Race	Mean MCMV score (BLUE value)*	Elite line used in breeding cross	Number of F_3_ lines derived
VERA179	Mexico	Tuxpeño	1.22	CML537	33
SCRO1	Virgin Islands	Saint Croix	1.25	CML537	99
RDOM169	Dominican Republic	Tuson, Tuzon	1.26	CML537	77
ECUA327	Ecuador	Cuban Yellow Dent	1.26	CML537	54
PERU558	Peru	Arizona	1.26	CML537	7†
VERA203	Mexico	Tuxpeño	1.27	CML537	18†
BRVI2	British Virgin Isl.	Saint Croix	1.28	CML549	147
PUER2	Puerto Rico	Chandelle	1.28	CML550	130
CUBA9	Cuba	Cuban Flint	1.29	CML550	106
HAIT44	Haiti	Haiti White	1.31	CML494	48
OAXA106	Mexico	Conico	1.36	CML549	44
CML494	CIMMYT line (Resistant check)	–	1.15	–	–
CML550	CIMMYT line (Resistant check)	–	1.47	–	–
CML334	CIMMYT line (Susceptible check)	–	1.64	–	–
CML228	CIMMYT line (Susceptible check)	–	2.11	–	–
*Trial Mean*			*1.68*		
*H^2^*			*0.78*		

* Mean MCMV score based on data from three trials; responses recorded on a 1 to 3 scale (1 = no visual MCMV symptoms; 2 = a few visual MCMV symptoms; 3 = severe MCMV symptoms).

† Not enough F3 lines derived for further testing.

### 3.3 Molecular breeding for developing MLN-resistant maize germplasm

Besides deriving MLN-tolerant/resistant inbred lines and hybrids through conventional breeding, molecular marker-based analyses assisted us in: a) understanding the genetic architecture of resistance to MLN and its causal viruses; b) identifying genomic regions and molecular markers associated with MLN/MCMV resistance, which are further used as trait-linked markers to improve the MLN resistance in breeding pipelines, including a forward breeding strategy to select lines for MLN resistance during early generations of breeding. Further, this knowledge assisted the breeders in designing and implementing an appropriate breeding strategy to develop MLN resistant lines/hybrids.

#### 3.3.1 Genetic architecture of MLN, MCMV and SCMV resistance in maize

Several studies reported the genetic basis of resistance to SCMV as controlled by two major genes *scmv 1* (at 14 to 15 Mb on chromosome 6) and *scmv 2* (at 133 Mb on chromosome 3) and other minor effect quantitative trait loci or QTL (Lübberstedt et al., 2006; Ingvardsen et al., 2010; Tao et al., 2013; Zambrano et al., 2014). However, nothing much was known about genes/genomic regions influencing MLN and MCMV resistance. To dissect the genetic architecture of resistance to MLN and MCMV, the CIMMYT Maize Program initiated discovery work on two association mapping (AM) panels, namely DTMA (Drought Tolerant Maize for Africa) and IMAS (Improved Maize for African Soils), through a genome-wide association study (GWAS; Gowda et al., 2015; Sitonik et al., 2019). The IMAS-AM and DTMA-AM panels comprised 380 and 235 lines, respectively, broadly representing the tropical/subtropical maize genetic diversity, including germplasm derived from breeding programs targeting tolerance to drought, soil acidity, low N, resistance to insects and pathogens (Wen et al., 2011). All 615 lines were evaluated against MLN under artificial inoculation for three crop seasons at Naivasha, Kenya. Both the panels were genotyped using genotyping-by-sequencing (GBS). Phenotypic variation was significant for MLN resistance, and heritability was moderate to high in both the panels.

In the IMAS-AM panel, we detected 18 significant marker–trait associations for MLN resistance. These significantly associated SNPs individually explained 8–10% of the total phenotypic variance, whereas together these explained 30% of the total proportion of phenotypic variance for MLN resistance (Gowda et al., 2015). In the DTMA-AM panel, we detected six significant marker–trait associations which individually explained 14–18% of the total phenotypic variance and together explained 37% of the total proportion of phenotypic variance for MLN resistance. Overall, the study revealed that MLN resistance is controlled by multiple loci with small to medium effects (Gowda et al., 2015). Further, to validate the GWAS results and identify new sources of resistance, we used four biparental populations and applied linkage mapping and joint linkage association mapping (JLAM) approaches (Gowda et al., 2018). Linkage mapping revealed three major QTL on chromosomes 3, 6, and 9 that were consistently detected in at least two of the four populations. These genomic regions coincided with previously reported potyvirus resistance loci, which we further fine-mapped and found new markers in tropical germplasm (Gowda et al., 2015). Because the trait used for evaluation in the study was MLN and not SCMV, the genomic regions may either represent resistance to only SCMV or for both SCMV and MCMV; this needs to be further studied. Phenotypic variance explained by a single QTL in each population ranged from 3.9% to 43.8%. The QTL identified on chromosomes 3, 5, 6, and 9 through JLAM were consistent with the QTL identified by linkage mapping. The study validated genomic regions identified through GWAS, and further identified new genomic regions associated with MLN resistance. Both studies revealed the presence of major effect QTL on chromosomes 3, 6, and 9 which are potential candidates for marker-assisted breeding to improve MLN resistance. Genomic prediction (GP) was also found to be another well-suited approach to capture maximum variation for the MLN resistance, including variation contributed by both the major and minor effect QTL (Gowda et al., 2018).

Jones et al. (2018) first reported a major QTL on chromosome 6 (at 157 Mb) influencing resistance to MCMV, which is recessively inherited in a set of F_2_ populations. This QTL was further confirmed in five F_2_ and three F_3_ populations and fine mapped the QTL region to find two strongly linked flanking markers (Murithi, 2019). Sitonik et al. (2019) studied the genetic basis of MCMV resistance using phenotypic and genotypic data (292,000 SNPs) on IMAS-AM panel (380 lines) and three DH populations and combining the data from GWAS, JLAM and linkage mapping. For all populations, phenotypic variation was significant, and heritability was moderate to high. Linkage mapping revealed 13 QTL for MCMV resistance and 12 QTL for MLN resistance. One major effect QTL *qMCMV3-108/qMLN3-108* was consistent across populations for both MCMV and MLN resistance, and was identified as a potential candidate for improving MCMV/MLN resistance. Another major effect QTL *qMCMV6-17/qMLN6-17* was consistent across MCMV and MLN resistance, with >27% of phenotypic variance explained. Most of GWAS-identified SNPs on chromosome 1, 3, 6 and 7 were within the confidence interval of the QTL detected through linkage mapping and JLAM. Overall, the study revealed that MCMV resistance is controlled by a few major and many minor effect loci. Two separate 10-SNP panels have been developed to use in forward breeding through the High Throughput Genotyping (HTPG) Platform, an initiative funded by the BMGF and the CGIAR Excellence in Breeding (EiB) Platform.

The CIMMYT Maize Program, after screening >100,000 germplasm entries at the MLN Screening Facility at Naivasha, identified two exotic maize lines (KS23-5 and KS23-6) as strong sources of MLN resistance. These lines were derived from synthetic populations developed by Kasetsart University, Thailand. KS23 is a broad-based synthetic line developed by intermating 26 inbred lines which combined well with a strain of Suwan1 (Jampatog et al., 2010). KS23 contains approximately 35% temperate germplasm and was designed to be a counterpart of Suwan1 in the hybrid breeding program. Although the exact origin of the MLN resistance in KS23 is not known, KS23-5 and KS23-6 serve as excellent trait donors. By using these KS lines, four F_2_ populations and three F_3_ populations were developed and genotyped with DART Seq markers. A major QTL for MLN resistance was independently identified in KS23-6 by Jones et al. (2018) in a cross between KS23-6/Oh28, and by CIMMYT in three separate populations (KS23-5/CZL0005, KS23-5/CML545, KS23-6/CZL03018). The recessively inherited QTL (*qMLN_06.157*) from KS23-6 (Fig. 1B) was further validated in an additional seven bi-parental populations and has been recently fine-mapped to a ∼6 kb interval in collaboration with Corteva Agriscience (Kanwarpal Dhugga and Mark Jung, personal communication).

#### 3.3.2 Marker-assisted backcrossing and forward breeding for MLN resistance

Most of the elite Africa-adapted CIMMYT lines used in commercial products were highly susceptible to MLN, and commercial hybrids in Kenya showed 70–100% yield loss under heavy MLN pressure. Twenty-five recurrent parent (RP) lines (from CIMMYT) were prioritized based on their parentage in the commercial maize hybrids in eastern and southern Africa or in the advanced breeding pipeline. RP and donor parent (DP) lines were organized according to heterotic groups (A and B) so that DP alleles would be deployed uniquely within each heterotic group (HG). A three-season continuous nursery was established in Kenya. Using markers linked to putative QTL from six donor lines (Gowda et al., 2015, 2018), a set of 2–4 loci per project (RP x DP combination) were targeted for introgression. BC_4_F_3_ lines were testcrossed with MLN-tolerant testers and evaluated to assess efficacy of the introgressions in the presence of MLN, and equivalency of the versions with the respective RPs in the absence of MLN. New converted versions were selected for advancement from 22 of 25 RP backgrounds in the MABC pipeline based on data from two locations with severe MLN infection, and two locations with no MLN pressure. One to six versions were advanced per RP with selected converted versions ranging from 1.0 to 3.1 t/ha yield advantage over the respective control (original RP testcrossed to a common MLN-tolerant tester). Mean grain yield of advanced lethal necrosis tolerant (LNT) versions was 5.3 t/ha, compared to 3.1 t/ha mean grain yield of the original RP control hybrids. Genetic analysis to identify which target alleles had greatest efficacy is in progress.

**Fig. 3:**
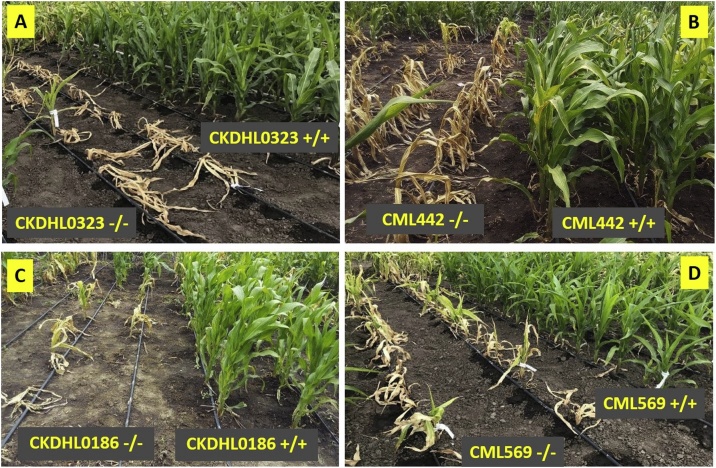
Responses of MLN-resistant versions vis-à-vis the recurrent parents (elite but MLN-susceptible lines) 30 days after first artificial inoculation at the MLN screening facility in Naivasha, Kenya. The MLN-resistant versions have *qMLN_06.157* from KS23-6 (donor parent), transferred through marker-assisted backcrossing.

A second major MABC pipeline is ongoing to bring *qMLN_06.157* (from KS23 background) into 30 Africa-adapted HG-A and HG-B lines of importance. Since the favorable allele in this case is recessive, it is required on both sides of the hybrid pedigree. In order to minimize the impact of unintended inbreeding, the primary strategy has been to deploy the allele into HG-B through a backcrossing approach until converted versions of HG-B lines can be used as breeding parents. *qMLN_06.157* has proven efficacious in multiple near-isogenic inbred backgrounds (Fig. 3). BC_3_ or BC_4_ versions of elite African lines were testcrossed and the hybrid trials were evaluated in 2019.

In parallel with the objective of capturing both major effect and minor effect QTL to develop comprehensive MLN resistant lines, the CIMMYT team in Africa also initiated genomic selection with four resistant x resistant (R x R) F3 populations. The first cycle of phenotype-based selection and second cycle of marker-based selection has been completed. The third cycle of marker-based selection is ongoing, and the lines are being validated in 2019 for their responses under MLN artificial inoculation at Naivasha.

A forward breeding panel incorporating *msv1* and *qMLN_06.157*, and an MABC panel which includes tightly linked target markers as well as additional nearby markers were used. A total of 3500 F_4_ ears from 39 HG-A source populations carrying *qMLN_06.157* were delivered to CIMMYT maize breeders in 2017. Six of the enriched F_4_ populations had a substantial shift towards resistance from the expected distribution. *qMLN_06.157* has been used to screen more than 20,000 DH and pedigree lines, and >5000 F2 lines to date. Three SNPs/haplotypes previously identified for MLN resistance (*qMLN03.133, qMLN03.146* and *qMLN06.21*) are also being used in forward breeding through the HTPG platform by the Natural Agricultural Research Organization (NARO)-Uganda, Ethiopian Institute of Agricultural Research (EIAR)-Ethiopia, and some seed companies in Uganda, Kenya and Zimbabwe.

### 3.4 Gene editing for MLN resistance

The molecular mechanism that explains how two different viruses (MCMV and SCMV) together cause the MLN disease is not yet known. Regardless, if a large-effect QTL for MLN resistance is identified and validated, this could be fine-mapped to identify the underlying gene. Gene editing could then be used to recreate appropriate favorable polymorphisms in the MLN-susceptible maize lines. However, the challenge will be to edit the exact causal allele in the susceptible but elite maize lines, especially parents of popular commercial hybrids in Africa, and create resistant versions directly.

Backcross breeding involves crossing an elite, commercial line (as a recurrent parent) to a donor parent (with MLN resistance) and then backcrossing over many cycles to recover the recurrent parent genome while introgressing the trait of interest from the donor parent. Backcrossing is a resource-intensive and time-consuming process. Even after elimination of a substantial proportion of the donor genome in the converted elite line, some of the not-so-desirable donor genes continue to be present, leading to unpredictable effects on performance. As most of the hybrids grown in Africa are three-way crosses, all three lines for each hybrid would require introduction of the recessive allele for resistance against MLN. CIMMYT focused on a large-effect QTL for MLN resistance from an exotic maize line, KS23-6 (Jones et al., 2018), which was validated in a number of populations generated by crossing this line to CIMMYT lines. In partnership with Corteva Agriscience and under a grant from BMGF, CIMMYT has fine-mapped this QTL to a 6 kb genetic interval (Kanwarpal Dhugga and Mark Jung, personal communication). Recessive inheritance suggests either a loss of function, which could result from an inactive form of the corresponding protein required by the virus for its replication or movement, or an altered protein sequence with a different conformation, which the virus is unable to recognize. Viruses are known to expropriate their host’s protein translation machinery for the translation of their own proteins.

The field of gene editing has progressed through several phases starting with oligo-mediated gene editing in the 1980s (Carroll, 2017). The main hurdle in widespread adoption of gene editing was the low frequency of the edited events, which makes progress painstakingly slow. A relatively new technique, clustered regularly interspersed short palindromic repeats (CRISPR) and CRISPR-associated protein 9 (Cas9), together referred to as CRISPR-Cas9, has revolutionized the field of gene editing because of its ease of use and a high success rate (Carroll, 2017). The CRISPR-Cas9 system generally leads to three different outcomes: site-directed nuclease-1 (SDN1), where after a cut by the CRISPR-Cas9 of the host DNA non-homologous end joining (NHEJ) introduces random mutations during repair leading to gene inactivation in some instances; SDN2, which involves template-mediated sequence alteration to change the gene function; and SDN3, where a DNA fragment is inserted at a precise location in the genome (Podevin et al., 2013; Savitashev et al., 2015).

A potential challenge in gene editing is the ability to transform elite maize lines that are parents of the MLN-susceptible commercial hybrids in Africa. This hurdle has been recently overcome by including cell cycle genes in the transformation vector (Lowe et al., 2016, 2018). The partnership between Corteva Agriscience and CIMMYT has used this technology to successfully transform four elite CIMMYT inbred lines, which are parental lines of two popular, heat- and drought-tolerant commercial hybrids in eastern Africa. All four lines and their hybrids are susceptible to MLN, however. The ability to transform the tropical lines has paved the way to use SDN1 or SDN2 gene editing approaches directly in the commercial lines. After two outcrosses to the unedited parent and simultaneous screening for unintended changes in the genome with highly sensitive molecular tools, the lines are ready to test in the field (Zastrow-Hayes et al., 2015). The major QTL for MLN resistance identified in KS23-6 could act as a background source of resistance or could be directly created in other genetic backgrounds to further fortify their partial MLN resistance.

Deletion of various parts of the 6 kb genetic interval where the KS23-6 QTL for MLN resistance resides using CRISPR-Cas9 is currently underway to validate the causal polymorphism. Once validated, the causal gene will be directly edited in the aforementioned four CIMMYT lines followed by reconstitution of the two, original three-way hybrids, which will then be tested for performance under normal growing conditions in Kiboko and under artificial inoculation in CIMMYT’s MLN screening facility at Naivasha.

## 4 Deployment of MLN-tolerant/resistant maize hybrids in Africa

When CIMMYT initiated maize germplasm screening under MLN artificial inoculation at Naivasha in 2013, a few CIMMYT pre-commercial hybrids showed MLN tolerance (MLN score of 5.0 on a 1–9 scale). CIMMYT provided initial seed, descriptor data and seed producibility information to the partners for evaluation under fast-tracked National Performance Trials (NPTs) in Kenya, Tanzania and Uganda, for varietal release and commercialization. Thus, four first-generation CIMMYT-derived MLN-tolerant hybrids (each with a mean MLN severity score of 5.0–6.0 on a 1–9 scale, as compared to commercial MLN-susceptible hybrids with severity scores above 7), were released by public/private sector partners in the region (1 in Uganda; 2 in Kenya; 1 in Tanzania) during 2013–2014 (Table 1).

CIMMYT breeders subsequently developed second-generation MLN-resistant hybrids (with MLN scores of 3.0–4.0 on a 1–9 scale); these hybrids were evaluated under NPTs, leading to the release of several hybrids during the next five years, especially in Kenya (Table 1). Although two years of NPT is a must for release of new hybrids in most of the East African countries, the period required for commercialization after release of a new hybrid differs among the countries. For instance, Bazooka and H6506 were recommended for release in Uganda and Kenya, respectively, in 2013. In 2014, NASECO (a seed company based in Uganda) produced 0.5 tons certified seed of Bazooka for promotion using the initial parental seed provided by CIMMYT and National Agriculture Research Organization (NARO). By 2018, certified seed production of “Bazooka” reached nearly 1500 tons, and the hybrid has been successfully marketed by NASECO in Uganda, D.R. Congo and Burundi. In Kenya, on the other hand, the new hybrid, H6506, had to first complete the DUS (Distinctness, Uniformity, Stability) testing, followed by bulking of the breeder seed under the supervision of the regulatory agency, KEPHIS, before the variety reached certified seed production and marketing levels. Thus, certified seed production and commercialization of H6506 (drought tolerant and MLN tolerant hybrid) was initiated in 2018. In 2019, Kenya Seed Company produced more than 300 tons of certified seed of H6506 for commercialization in 2020. The second-generation MLN-resistant hybrids are expected to be commercialized from 2020 onward in East Africa.

## 5 Diagnostics for MLN-causing viruses

Development and deployment of sensitive and efficient diagnostics for MLN-causing viruses is essential for disease surveillance at various levels, besides epidemiological studies and screening and selection of MLN-resistant cultivars. Availability of appropriate diagnostic tools also enables free and safe exchange of disease-free germplasm across international borders, in stride with the increased globalization and proliferation of free trade agreements.

There are numerous serological and molecular diagnostic assays that are available for detection of MCMV and SCMV from leaf tissues. However, serological assays may not necessarily produce consistent results for SCMV due to its high diversity (Braidwood et al., 2019). There are commercially available immunostrip kits which are easy to use, cost-effective, and offer quick and accurate results for MCMV detection. The immunostrips have been used widely for monitoring MCMV within seed production fields in eastern Africa (https://mln.cimmyt.org/mln-diagnostic-techniques/#immunostrips).

Advanced molecular tools may help overcome some of the shortcomings of ELISA in terms of sensitivity, but not on the basis of cost effectiveness. The use of RT-PCR enables amplification of target RNA of the virus and is proven to have greater sensitivity than ELISA. Next generation sequencing (NGS) technology, first used for plant virus detection in 2009 (Adams et al., 2013), has since been used to identify the causes of various crop diseases and for studying the molecular diversity of the viruses. However, such technology is often not available locally or routinely applicable in the eastern Africa region due to high cost of equipment, reagent availability, operational costs, service support, and limited capacities in the field of bioinformatics. The introduction of simpler, table-top sequencers, such as MiSeq (Illumina) and Ion Torrent (Life Technologies), has made the NGS technology easier to access, bringing down both the capital cost of the equipment and per sample cost of the reagents. Despite this, the technology is still only available in specialist diagnostic laboratories (Boonham et al., 2014).

The challenge in field-based MCMV detection is making available cheap, user-friendly and sensitive diagnostic tools that can work in remote areas, with limited or no equipment and technical skills. Despite availability of several sophisticated technologies for MLN virus diagnostics, there are simple, cost-effective and reliable diagnostic tools such as immunostrips, which can be routinely used on leaf samples in field-based diagnosis, including MLN/MCMV surveillance work in farmers’ fields and seed production fields. Under a USAID funded Project on MLN Diagnostics and Management in Africa, protocols have been optimized for leaf sampling in the farmers’ field and maize seed production fields for immunostrip-based MCMV diagnosis (Mezzalama et al., 2015; https://mln.cimmyt.org/). NPPO personnel in several countries in eastern, southern and West Africa have been trained for routine use of commercially available MCMV immunostrips. Field-based surveillance and monitoring activities, coupled with MCMV immunostrip based analyses, have been implemented over the last four years across eastern and southern Africa (Table 5).

**Table 5:** MCMV immunostrip data summary from 2014 to 2019 based on surveys undertaken in eight countries in eastern and southern Africa. All the data from 2016 onwards were collected using standardized diagnostics protocols. Historical data from 2014 and 2015 are included only for comparison.

Country	MCMV data	Year					
		2019	2018	2017	2016	2015	2014
**Ethiopia**	**Total Surveys**	**420**	**301**	**263**	No data	No data	No data
	MCMV + ve	146	19	71	No data	No data	No data
	MCMV -ve	274	282	192	No data	No data	No data
	**% MCMV + ve**	**35**	**6**	**27**	No data	No data	No data
**Kenya**	**Total Surveys**	**350**	**198**	**200**	No data	**137**	**105**
	MCMV + ve	56	36	7	No data	54	24
	MCMV -ve	294	162	193	No data	83	81
	**% MCMV + ve**	**16**	**18**	**4**	No data	**39**	**23**
**Tanzania**	**Total Surveys**	**66**	No data	No data	**67**	**141**	No data
	MCMV + ve	22	No data	No data	13	97	No data
	MCMV -ve	44	No data	No data	54	44	No data
	**% MCMV + ve**	**33**	No data	No data	**19**	**69**	No data
**Rwanda**	**Total Surveys**	**477**	**176**	No data	No data	**399**	No data
	MCMV + ve	38	19	No data	No data	167	No data
	MCMV -ve	439	157	No data	No data	232	No data
	**% MCMV + ve**	**8**	**11**	No data	No data	**42**	No data
**Uganda**	**Total Surveys**	**141**	**157**	**69**	No data	**245**	**358**
	MCMV + ve	24	147	18	No data	14	23
	MCMV -ve	117	10	51	No data	231	335
	**% MCMV + ve**	**17**	**94**	**26**	No data	**6**	**6**
**Malawi**	**Total Surveys**	**373**	**173**	**312**	**207**	No data	No data
	MCMV + ve	0	0	0	0	No data	No data
	MCMV -ve	373	173	312	207	No data	No data
	**% MCMV + ve**	**0**	**0**	**0**	**0**	No data	No data
**Zambia**	**Total Surveys**	**240**	**179**	**335**	**128**	No data	No data
	MCMV + ve	0	0	0	0	No data	No data
	MCMV -ve	240	179	335	128	No data	No data
	**% MCMV + ve**	**0**	**0**	**0**	**0**	No data	No data
**Zimbabwe**	**Total Surveys**	**265**	**280**	**284**	**223**	No data	No data
	MCMV + ve	0	0	0	0	No data	No data
	MCMV -ve	265	280	284	223	No data	No data
	**% MCMV + ve**	**0**	**0**	**0**	**0**	No data	No data
	**Total Surveys**	**2332**	**1464**	**1463**	**625**	**922**	**463**

## 6 MLN monitoring and surveillance across Africa

MLN represents another example where a successful, large-scale surveillance system for an emerging transboundary disease has been developed as part of a rapid response mechanism led by a CGIAR center. This rapid spread, coupled with widespread cultivation of highly susceptible maize varieties and losses of maize crops in the farmer’ fields, was the catalyst to develop a coordinated MLN surveillance, monitoring and information dissemination network in SSA. MCMV was reported to be transmitted at a very low rate (less than 0.04%) through contaminated seed (Jensen et al., 1991). Rate of seed transmission of MCMV in Kenya from contaminated seed was found to be low at 0.059% (Kimani et al., unpublished data); this is comparable to the rate of seed transmission of MCMV reported by Jensen et al. (1991). Although the rates and relative importance of various purported transmission mechanisms of MLN (insect-vectors, contaminated seed, contaminated soil, mechanical means) have not been fully determined (Redinbaugh and Stewart, 2018), rigorous and continued surveillance and diagnosis are critical for tracking the disease and minimizing its spread on the continent. The surveillance and disease tracking for MLN were modelled on the successful initiative for wheat stem rust (Ug99) (Hodson et al., 2012; Park et al., 2011). Existing wheat rust surveillance systems and platforms were a key factor behind the ability to develop and implement MLN surveillance in a rapid manner. The MLN surveillance system has been developed by CIMMYT in partnership with NPPOs and Aarhus University, Denmark, under the USAID-funded MLN Diagnostics and Management Project. After its initial development in 2015, a fully operational regional MLN surveillance system is now in place.

**Fig. 4:**
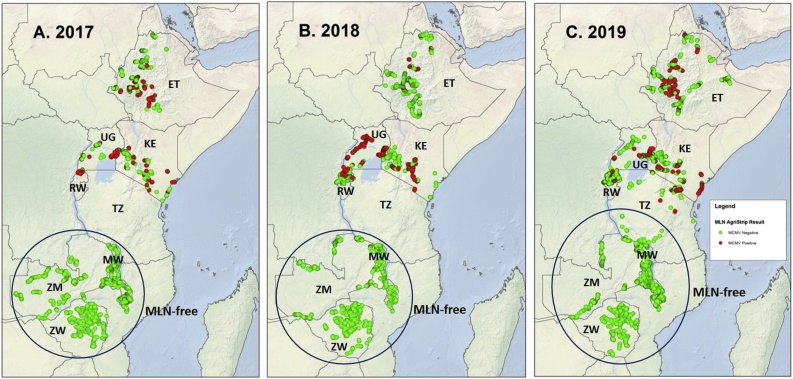
Results of MLN surveys (based on MCMV immunostrip data, coupled with evaluation for MLN symptoms, if any) undertaken by NPPOs in eastern and southern Africa, in partnership with CIMMYT, in **(A)** 2017 versus **(B)** 2018 versus **(C)** 2019.

Regional MLN surveillance using standardized protocols (https://mln.cimmyt.org/mln-status/protocols-survey-forms/) was carried in eight sub-Saharan countries, namely Kenya, Uganda, Tanzania, Rwanda, Malawi, Zambia and Zimbabwe. A set of standardized MLN surveillance, sampling and MCMV diagnostic protocols have been developed by CIMMYT and NPPO partners in 2015 (Mezzalama et al., 2015; https://mln.cimmyt.org/). The survey results provided updated information on the status of MLN, including the diagnostic symptoms and MCMV immunostrip data. Country-level survey data are summarized in Table 5. All surveys conducted since 2016 have used MCMV immunostrips to detect the presence of MCMV from a bulk sample of 6 young leaves per hectare, collected randomly using a staggered X pattern. Survey fields were selected at random every 10–20 km in maize growing areas. Fields sprayed with pesticide were not surveyed. All surveyed fields were geo-referenced using GPS. Field survey data from 2017, 2018 and 2019 are shown in Fig. 4. Recent surveys indicate that MCMV continues to prevail in eastern Africa. Kenya, Uganda, Rwanda, northern Tanzania and Ethiopia all detected the presence of MCMV in farmers’ fields (Table 5). However, no further spread to new countries has been detected and current survey data indicates the continued absence of MCMV/MLN in the southern highlands of Tanzania, Malawi, Zambia and Zimbabwe (https://mln.cimmyt.org/).

Incidence of MCMV in MLN-prevalent eastern African countries varied between countries and years (Table 5). No consistent patterns were observed among years or countries, although there was a possible indication that incidence of MCMV had decreased in Rwanda in recent years. Apparent increases in MCMV incidence (compared to the previous years) were detected in several countries – for example, Uganda in 2018, Kenya in 2018, and Ethiopia in 2019. Drivers behind the increased incidence of MCMV in Uganda and Kenya in 2018, and in Ethiopia in 2019, are currently unknown and need further research. In Kenya, the disease is still severe in Bomet, Kericho, Kajiado and Narok counties with incidences of over 40% and average MCMV severity of 6 on a 1–9 scale (Sitonik et al., 2019) (1: completely “clean” plants with no visible MCMV symptoms; 2: fine or no chlorotic specks, but no loss of plant vigor; 3: mild chlorotic streaks on emerging leaves; 4: moderate chlorotic streaks on emerging leaves; 5: chlorotic streaks and mottling throughout the plant; 6: intense chlorotic mottling throughout the plant, with necrosis of leaf margins; 7: excessive chlorotic mottling, mosaics, and leaf necrosis in most of the plant; 8: excessive chlorotic mottling, leaf necrosis, dead heart, and sometimes premature death of plants; and 9: complete plant necrosis, and sometimes even dead plants).

### 6.1 MLN monitoring tools and surveillance network

Given the transboundary nature of MLN, a standardized and harmonized monitoring system operating at the regional level was considered essential. A set of standardized MLN surveillance, sampling and diagnostic protocols have been developed by CIMMYT and NPPO partners since 2015. These surveillance and diagnostic protocols are now available (https://mln.cimmyt.org/) and are currently being used by NPPOs across SSA, especially in eastern and southern Africa, in their national MLN surveillance programs. Annual surveillance and monitoring programs are presently being implemented in eight countries – five MLN-prevalent countries in eastern Africa (Ethiopia, Kenya, Rwanda, Tanzania, and Uganda), and three major commercial maize-seed producing but MLN-free countries in southern Africa (Malawi, Zambia and Zimbabwe), with funding support from the USAID-East Africa Office.

Trained national surveillance teams are using an open-data kit (ODK)-based smartphone survey app to collect geo-referenced field survey data from the major maize-growing areas in each of the eight countries on an annual basis. Data collection is focused on MLN, but other important maize pest and diseases are also recorded. At each field site, visual observations are supported by standardized leaf sampling and *in-situ* testing using immunostrips for MCMV. MCMV is the target for field testing as this is the major virus that drives MLN expansion (Redinbaugh and Stewart, 2018). In addition, seed samples are collected from agro-dealers and import sites on an annual basis; seed sample site data are recorded using an ODK app and samples tested for MCMV using ELISA.

### 6.2 MLN surveillance data management toolbox

In collaboration with Aarhus University, Denmark, CIMMYT developed an MLN Surveillance Data Management Toolbox, an online data management system that supports field surveillance and seed surveys of MLN and other major maize diseases in SSA. The MLN toolbox enables centralized and secure management of standardized data for transboundary diseases at the continental level but with data managed, validated and published at the country level. Features of the MLN toolbox include: controlled access, secure storage in structured databases, data editing, data visualization through interactive maps and charts, and data export of country-specific data. Only when data has been checked and approved by authorized country managers does it enter into public domain data dissemination tools. The MLN toolbox represents an increasingly rich data resource on the status of MLN in SSA, with over 7000 field survey records from 8 countries and more than 400 seed survey records currently in the database.

## 7 Production and exchange of MLN pathogen-free commercial maize seed

While detection of MLN-causing viruses on contaminated seed may not necessarily lead to transmission of the disease to the next generation, from the phytosanitary perspective, it is important to evaluate the presence of MCMV and SCMV in commercial seed lots, especially those meant for exportation to countries where MLN/MCMV is not reported. In practice, keeping a commercial seed production field completely free from the MLN-causing viruses in areas where the disease is widely prevalent requires significant efforts and resources, but is important for protecting the food security, income and livelihoods of the resource-poor smallholder farmers. Testing for MLN viruses in the seed is also important for NPPOs to ensure that the seed shipped to other countries is devoid of the pathogens, especially to those countries in which MLN is not prevalent or reported.

During the early years of MLN outbreak in eastern Africa, most of the local/regional seed companies in the MLN-prevalent countries lacked necessary knowledge of the disease and its transmission, as well as protocols to produce MLN pathogen-free clean seed. It was, therefore, critical to develop and implement protocols for MLN pathogen-free seed production and exchange, not only from the MLN-prevalent areas to those where the disease is not prevalent but also within the areas where MLN is widely prevalent, and make these protocols widely accessible to commercial maize seed companies. Several strategies have been put in place in order to achieve this objective; these include development of comprehensive standard operating procedures (SOPs) and checklists for MLN pathogen-free seed production at various points along the seed value chain (https://mln.cimmyt.org/). Three consultative meetings, one each in Kenya, Tanzania and Ethiopia, organized jointly by CIMMYT, AATF and AGRA, including researchers, seed companies and extension agents helped in harmonizing the MLN virus-free seed production checklist and Standard Operational Procedures (SOPs).

Several training workshops were organized in the MLN-prevalent countries under the USAID MLN Diagnostics and Management Project i.e., Kenya, Uganda, Ethiopia, Tanzania and Rwanda. The trainings targeted seed companies, seed trade associations, contract growers, NARS institutions, regulatory and extension agencies for disseminating SOPs and MLN management checklist. A total of 574 participants from NPPOs and NARS institutions, 544 participants from commercial seed companies, and 2313 small-scale contract seed growers in eastern Africa were trained during 2016–2019 on the SOPs for MLN pathogen-free seed production. The course content included on-farm MLN diagnostics, disease scouting, leaf and seed sampling, and testing using immunostrips and ELISA. Immunostrip-based rapid diagnostic kits were procured and disseminated to NARS institutions and local seed companies in countries where MLN is widely prevalent to ensure their promotion within the seed companies and for strengthening internal quality control systems. More than 30 seed companies are presently implementing the MLN-free seed production checklist on a voluntary basis in Kenya, Uganda, Rwanda, Tanzania and Ethiopia. The public and private sector institutions, including extension agencies, were encouraged to use the MCMV immunostrips to test for the virus incidence in commercial seed production fields since some plants, especially the maize cultivars with high levels of tolerance to the disease may harbor the MLN viruses without showing the symptoms. The immunostrip results helped the seed companies to make informed decisions on MLN management practices, including identification and roguing of infected plants very early to reduce the risk of disease spread. On-farm visits were also made by partner institutions, especially AGRA and AATF, to ascertain the status of incidence of MLN viruses (especially MCMV), and to monitor voluntary implementation of harmonized SOPs and MLN management checklist. Various communication materials on MLN diagnostics and management (available through the MLN web portal; https://mln.cimmyt.org/) were developed and distributed to relevant seed stakeholders in MLN-prevalent countries as well as those where the disease is not prevalent or reported so far in Africa.

## 8 Awareness creation about MLN management, and MLN phytosanitary community of practice

MLN mitigation strategies require well-coordinated institutional efforts that effectively leverage expertise across multiple institutions. To achieve this, the MLN Phytosanitary Community of Practice (CoP) was established by CIMMYT in Africa in 2016, bringing together diverse partners, including phytosanitary and regulatory organizations, seed trade associations, NARS scientists, regional bodies etc. The forum currently has a membership of 62 individuals representing diverse organizations, including NPPOs, NARS, international centers, NGOs, and the commercial seed sector across sub-Saharan Africa. The objectives of the CoP are: a) to identify, gather, and seek agreement on the phytosanitary community requirements, especially for effective control of MLN in SSA; b) to provide a forum/platform for cooperation on activities where the CoP adds value to the existing initiatives; c) to share learning across borders on key aspects, such as standardized MLN diagnostics procedure(s), providing training on MLN diagnostics, expediting adoption of appropriate phytosanitary and diagnostic procedures, identifying/validating and deploying novel and low-cost MLN diagnostic protocols, etc.; d) to identify linkages and opportunities for collaborative strategic and technical projects related to MLN phytosanitation and diagnostics in SSA; e) to report on progress and provide updates of the projects and programs that have phytosanitary and diagnostics components related to MLN; and f) to provide information for the review of maize seed certification and import/export procedures in relation to MLN for formulation of appropriate SOPs. The online discussion platform is a potentially sustainable means to discuss topical issues, with discussion summaries posted on the MLN web portal (https://mln.cimmyt.org/).

As a part of the strategy to ensure wider dissemination of information and increase awareness among relevant stakeholders, CIMMYT and partners in Africa established an MLN Information Portal (https://mln.cimmyt.org/). This portal aims to be a single source of updated information on MLN in sub-Saharan Africa. The MLN portal provides access to research information (e.g., the availability of new MLN-tolerant/resistant germplasm), MLN Screening Facility updates, MLN surveillance status, communication products, and training course materials. The MLN Toolbox (data management system) is connected directly to the MLN Information Portal, enabling database-driven interactive maps and charts of surveillance data to be displayed automatically. User statistics for the MLN Information Portal indicate an increasing number of visitors with a near global distribution.

## 9 Concluding remarks

MLN management in sub-Saharan Africa is a complex challenge. Nevertheless, through extensive partnerships, scientific institutions have been able to respond rapidly to this serious threat to the food security, income and livelihoods of millions of smallholder farmers and their families in SSA. MLN management has been effectively addressed through several simultaneously-implemented strategies, including development and deployment of elite MLN tolerant/resistant varieties adapted to Africa; coordinated and synergistic multi-disciplinary efforts of various national and international institutions engaged in maize R&D in Africa; intensive awareness creation among stakeholders, and capacity building of relevant public and private sector institutions on MLN diagnostics and management; devising and implementing a checklist and standard operating procedures for MLN-free commercial seed production and exchange by the commercial seed sector; and strong engagement of the national plant protection organizations (NPPOs) on MLN surveillance across SSA.

Intensive engagement with the NPPOs across sub-Saharan Africa, especially major maize-growing countries in southern Africa and West Africa, have so far been successful in preventing the spread of MLN from the eastern Africa where MLN is widely prevalent. Since 2014, no new country in sub-Saharan Africa has reported incidence of MLN. Among the various interventions implemented by CIMMYT and partners, capacity building of relevant public and private sector institutions on MLN diagnostics and management, intensive awareness creation among the stakeholders, and systematic monitoring and surveillance have been particularly impactful. Despite this, there is no room for complacency because the disease is still prevalent in various countries in eastern Africa, including Ethiopia, Kenya, Rwanda, Tanzania, and Uganda. Intensive surveillance for MCMV/MLN, coupled with integrated disease management, need to be continuously implemented in countries where MLN is prevalent as well as those countries which have so far not reported MLN. Since a large majority of commercial maize varieties presently cultivated in eastern Africa are MLN-susceptible, these varieties do serve as “reservoirs” for MLN-causing viruses, especially MCMV. It is, therefore, important to adopt an integrated disease management approach (Redinbaugh and Stewart, 2018), including wider adoption of improved MLN-resistant maize varieties, especially second-generation hybrids, in both MLN-prevalent countries and countries where the disease is not yet reported or prevalent; understanding vector dynamics and implementing control measures; production and exchange of “clean” commercial maize seed with no seed contamination by MLN-causing viruses; avoiding maize monoculture and continuous plantings of maize throughout the year; maize crop rotation with compatible crops, especially legumes, that do not serve as hosts for MCMV; and continued efforts on MLN disease monitoring and surveillance across sub-Saharan Africa.

## Declaration of Competing Interest

None.
